# Single-Molecule FRET Detection of Sub-Nanometer Distance Changes in the Range below a 3-Nanometer Scale

**DOI:** 10.3390/bios10110168

**Published:** 2020-11-08

**Authors:** Heyjin Son, Woori Mo, Jaeil Park, Joong-Wook Lee, Sanghwa Lee

**Affiliations:** 1 Advanced Photonics Research Institute, Gwangju Institute of Science and Technology, Gwangju 61005, Korea; heyjingist@gist.ac.kr (H.S.); bboy716.wr@gmail.com (W.M.); wodlfdlgh@gist.ac.kr (J.P.); 2 Department of Physics and Optoelectronics Convergence Research Center, Chonnam National University, Gwangju 61186, Korea; leejujc@chonnam.ac.kr

**Keywords:** single-molecule FRET, fluorescence, FRET probe, conformational dynamics

## Abstract

Single-molecule fluorescence energy transfer (FRET) detection has become a key technique to monitor intra- and intermolecular distance changes in biological processes. As the sensitive detection range of conventional FRET pairs is limited to 3–8 nm, complement probes are necessary for extending this typical working range. Here, we realized a single-molecule FRET assay for a short distance range of below 3 nm by using a Cy2–Cy7 pair having extremely small spectral overlap. Using two DNA duplexes with a small difference in the labeling position, we demonstrated that our assay can observe subtle changes at a short distance range. High sensitivity in the range of 1–3 nm and compatibility with the conventional FRET assay make this approach useful for understanding dynamics at a short distance.

## 1. Introduction

Single-molecule fluorescence energy transfer (FRET) has been widely used to provide structural and dynamic features of biomolecules [1,2,3]. This method allows us to monitor biomolecular interactions occurring on the nanometer scale. However, conventional FRET provides only the distance changes in the range of 3–8 nm, which is insufficient to investigate the subtle conformational changes of many biological systems in real time.

To reach the detection range below the lower limit, single-molecule techniques for observing distance changes occurring at short distances have been developed and used to solve a wide range of biological problems [4,5,6,7,8,9,10,11]. For instance, single-molecule protein-induced fluorescence enhancement (smPIFE) [4] provides the ability to measure the distance changes between a fluorophore and a protein in the range of 0–3 nm, but it makes only a limited contribution to monitoring inter-molecular interactions between a protein and another partner molecule. Hence, this method could not measure intramolecular conformational changes of single proteins or molecular interactions of biomolecules except for proteins. In the same line of technical development, self-quenching between two identical TMRs (homo-FRET) was utilized for single-molecule measurements at a short distance range [5]. Although this approach has no limitations regarding the sample type, this method is significantly influenced by environmental conditions and instrumental noise owing to its non-ratiometric quantification, unlike single-molecule FRET, which makes the data analysis and data interpretation more complicated.

**Figure 1:**
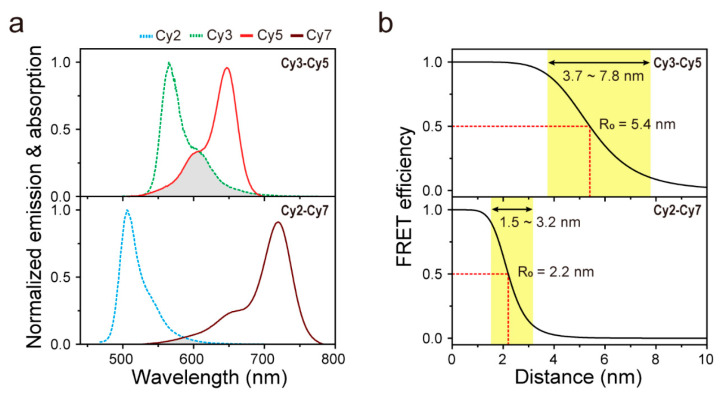
Selection of fluorescence energy transfer (FRET) pair for use at a short distance range. (**a**) Comparison of spectral overlaps between donor emission (dashed lines) and acceptor absorption (solid lines) spectra for Cy3–Cy5 pair (upper panel) and Cy2–Cy7 pair (lower panel). The emission and absorption spectra curves were obtained from the measurements. (**b**) FRET efficiencies of Cy3–Cy5 and Cy2–Cy7 pairs as a function of inter-dye distances. These curves were predicted from calculated R_0_ values.

In this study, we demonstrate that single-molecule FRET assay can be used to observe the subtle conformational changes over a short distance, in contrast to conventional FRET, by employing a cyanine dye pair (Cy2–Cy7), which has an extremely small spectral overlap between donor emission and acceptor absorption (Figure 1a). Small spectral overlap between donor emission and acceptor absorption has generally been considered to be undesirable for FRET probes [12,13], and hence, these dye pairs have rarely been used in single-molecule FRET measurements. Here, however, by actually exploiting this drawback, we propose a suitable FRET pair for measuring distance changes at a short distance range.

## 2. Materials and Methods

### 2.1. Characterization of FRET Pairs

To estimate the performance of the Cy2–Cy7 pair, we first compared the theoretical FRET range of this pair with that of a conventional FRET pair, Cy3–Cy5. To obtain the FRET ranges, Förster distances (*R*_0_) for both dye pairs were calculated using the following Equation:

*R*_0_^6^ = (0.529 ∙ *κ*^2^ ∙ *Φ_D_* ∙ *J*(*λ*))/(*N_A_* ∙ *n*^4^),

where *κ^2^* is the dipole orientation factor, *Φ_D_* is the fluorescence quantum yield of the donor, *J(λ)* is the spectral overlap integral of donor emission and acceptor absorption, *N_A_* is Avogadro’s number and *n* is the refractive index of the medium [14]. For this calculation, we used the quantum yields of donor fluorophores provided by the manufacturer (12% for Cy2 and 16% for Cy3) and assumed that *κ^2^* is 2/3 for freely rotating dyes. *J(λ)* was calculated from the measured donor emission and acceptor absorption spectrum. As a result, we determined *R_0_* values for Cy3–Cy5 and Cy2–Cy7 pairs as 5.4 and 2.2 nm, respectively. Based on the *R_0_* value of each FRET pair, we obtained the FRET efficiency (*E*) curves of both pairs as a function of the inter-dye distances (R) according to the relation *E = 1/(1 + (R/R_0_)*^6^, as shown in Figure 1b. These curves clearly show that the ability of the Cy2–Cy7 pair is sufficient to extend the working range, which will make it possible to monitor the distance changes occurring within the range below the lower limit of conventional FRET (~3 nm).

### 2.2. Preparation of DNA and Single-Molecule FRET Experiments

For single-molecule FRET measurements, high performance liquid chromatography (HPLC)-purified DNAs were purchased from Integrated DNA Technologies (Coralville, IA, USA) and labeled with Cy2, Cy3, Cy5 or Cy7 NHS-ester dyes (GE healthcare Life Sciences, Piscataway, NJ, USA) at the amine group of an internal amino modifier (dTC6). Then, DNA strands were annealed by slowly cooling down the biotinylated strand labeled with an acceptor (Cy5 or Cy7) and non-biotinylated strand with a donor (Cy3 or Cy2) from 95 °C to 4 °C in a buffer containing 10 mM Tris-HCl (pH 8.0) and 50 mM NaCl.

The prepared DNA duplexes were immobilized on the PEG-coated quartz slide via a streptavidin-biotin interaction. Single-molecule fluorescence images were taken by a home-built prism-type total internal-reflection microscope under the following buffer condition: 10 mM Tris-HCl (pH 8.0), 50 mM NaCl and the oxygen scavenging system (0.4% (*w*/*v*) glucose (Sigma, St Louis, MO, USA), 1% (*v*/*v*) Trolox (Sigma, St Louis, MO, USA), 1 mg/mL glucose oxidase (Sigma, St Louis, MO, USA) and 0.04 mg/mL catalase (Roche, Basel, Switzerland)) [2]. Cy2 or Cy3 was excited by a blue (473 nm) or a green (532 nm) laser. Fluorescence signals from Cy2–Cy7 or Cy3–Cy5 were collected by a water immersion objective lens (UPlanSApo 60x; Olympus, Tokyo, Japan), filtered through a 488-nm long-pass filter (LP02-488RE-25; Semrock, Rochester, NY, USA) for Cy2–Cy7 pair or a 540-nm long-pass filter (LP03-532RU-25; Semrock, Rochester, NY, USA) for Cy3–Cy5 pair, separated with a dichroic mirror (635dcxr; Chroma, Bellows Falls, VT, USA) and imaged onto an electron-multiplying charge coupled device (EM-CCD) camera (Ixon Ultra DU897U; Andor, Belfast, UK). The emission signals were split into two detection channels (Cy3–Cy5 or Cy2–Cy7), and were simultaneously recorded in real-time as one movie file.

To obtain time traces of donor and acceptor signals from the recorded movie, we first selected the co-localized donor and acceptor spots to exclude partially labeled molecules and extracted single-molecule time traces of fluorescence intensities from the selected spots. At least a hundred time traces were obtained from one movie file. Each time trace represents intensity profiles of a single FRET pair attached to an individual double-stranded DNA molecule. This converting process was performed by a custom script written in IDL (Exelis, Boulder, CO, USA).

Before estimating an accurate FRET efficiency, fluorescence signals should be corrected in three steps: (1) background subtraction, (2) correction for leakage between the detection channels and (3) correction for differences in detection efficiencies and emission quantum yields between fluorophores. These corrections were conducted in a straightforward manner using single fluorophores photobleaching events that exhibit a single-step intensity drop for an individual dye. First, backgrounds of donor and acceptor channels were determined by averaging their intensities of dozens of molecules when both donor and acceptor fluorophores were photobleached. After background subtraction, the leakage of donor emission into an acceptor channel was obtained by averaging the ratio of the donor fluorescence signal detected in the acceptor channel to that of the donor fluorescence signal detected in donor channel when the acceptor fluorophore was photobleached. Next, the correction that accounts for differences in detection efficiencies (*η_D_* and *η_A_*) and quantum yields (*ϕ_D_* and *ϕ_A_*) of donor and acceptor should be considered. In principle, FRET efficiency is expressed as $E={\left[1+\frac{I_{D}^{0}\cdot \phi_{A}}{I_{A}^{0}\cdot \phi_{D}}\right]}^{-1}$, where $I_{D}^{0}$ and $I_{A}^{0}$ are true donor intensity and sensitized emission intensity of acceptor in the presence of energy transfer, respectively. $I_{D}^{0}$ and $I_{A}^{0}$ can be reduced to measured intensities of the donor (*I_D_*) and acceptor (*I_A_*) by factors of *η_D_* and *η_A_* ($I_{D}=\eta_{D}\cdot I_{D}^{0}$ and $I_{A}=\eta_{A}\cdot I_{A}^{0}$). Therefore, we can rewrite FRET efficiency as $E={\left[1+\gamma \frac{I_{D}}{I_{A}}\right]}^{-1}$, where γ is $\eta_{A}\phi_{A}/\eta_{D}\phi_{D}$. The correction factor γ was determined by averaging the ratio of change in acceptor intensity to change in the donor intensity ($\gamma =\Delta I_{A}/\Delta I_{D}$) during acceptor photobleaching events occurred in our time traces of fluorescence intensity from dozens of molecules. After applying γ correction, fluorescence intensity of each fluorophore is normalized. In our experiments, γ values were obtained as 1 for Cy3–Cy5 pair and 2.5 for Cy2–Cy7 pair, respectively.

Finally, we obtained time courses of FRET efficiency E of each molecule from its donor and acceptor intensities (*I_D_* and *I_A_*) according to the expression *E* = [1 + *γ I_D_*/*I_A_*]^−1^. All data processing was conducted using custom scripts written in Matlab (Mathworks, Sherborn, MA, USA).

## 3. Results and Discussion

### 3.1. Model Prediction

**Figure 2:**
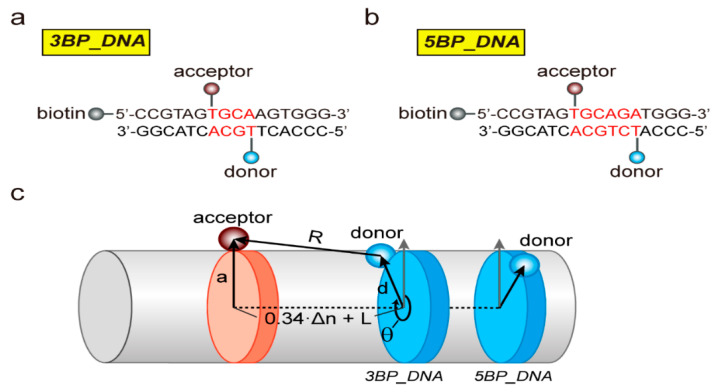
Structural model prediction for determining inter-dye distances. (**a**,**b**) DNA constructs, 3BP_DNA (**a**) and 5BP_DNA (**b**), used for the experiments. The labeling sites for conjugating donor and acceptor dyes are indicated. (**c**) Inter-dye prediction using cylindrical model for helical geometry of the double-stranded DNA used in measurements.

To demonstrate the ability to use the Cy2–Cy7 pair for single-molecule FRET measurements experimentally, we prepared two DNA duplexes, 3BP_DNA and 5BP_DNA, which have different labeling positions, as depicted in Figure 2a,b. The DNA sequences were randomly selected such that only canonical B-form DNA duplexes were generated. To test whether any possible undesired structures of DNA other than the canonical B-form DNA duplex could be formed, we used a DNA oligo analyzer tool to obtain Gibbs free energies of predicted DNA structures and did not find the other DNA structures with a high probability. These two DNA constructs have the same labeling positions for the acceptor fluorophore, but different positions for the donor fluorophore, such that different inter-dye distances are implemented. To predict the inter-dye distance of each DNA construct, we used a cylindrical model for double-stranded DNA as shown in Figure 2c [15,16,17,18,19]. For this structural model prediction, we estimated the inter-dye distance, *R*, using the following Equation:

*R* = *√*((0.34 ∙ Δ*n* + *L*)^2^ + *d*^2^ + *a*^2^ – 2 ∙ *d* ∙ *a* ∙ *cosθ*),

where Δ*n* is the number of base pairs between donor and acceptor dyes, *L* is the axial separation of the dyes for Δ*n* = 0, *d* and *a* are the radial distances of the donor and acceptor dyes from the helical axis, respectively, *θ* (*θ =* 36°∙Δ*n*+*φ*) is the rotation angle between the dyes and *φ* is the angular separation of the dyes for Δ*n* = 0. Based on DNA constructs used in this study, we assumed *L =* 0, *d = a =* 2 nm and *φ =* 240°. According to this model, the distances between donor and acceptor fluorophores in the two DNA constructs were estimated to be 1.10 (3BP_DNA) and 2.62 nm (5BP-DNA), respectively. To test whether the use of the Cy2–Cy7 pair in single-molecule FRET measurements differentiates these two DNA constructs, unlike the conventional Cy3–Cy5 pair, we prepared the DNA duplexes labeled with each FRET pair and performed single-molecule FRET measurements.

### 3.2. Experimental Demonstration

**Figure 3:**
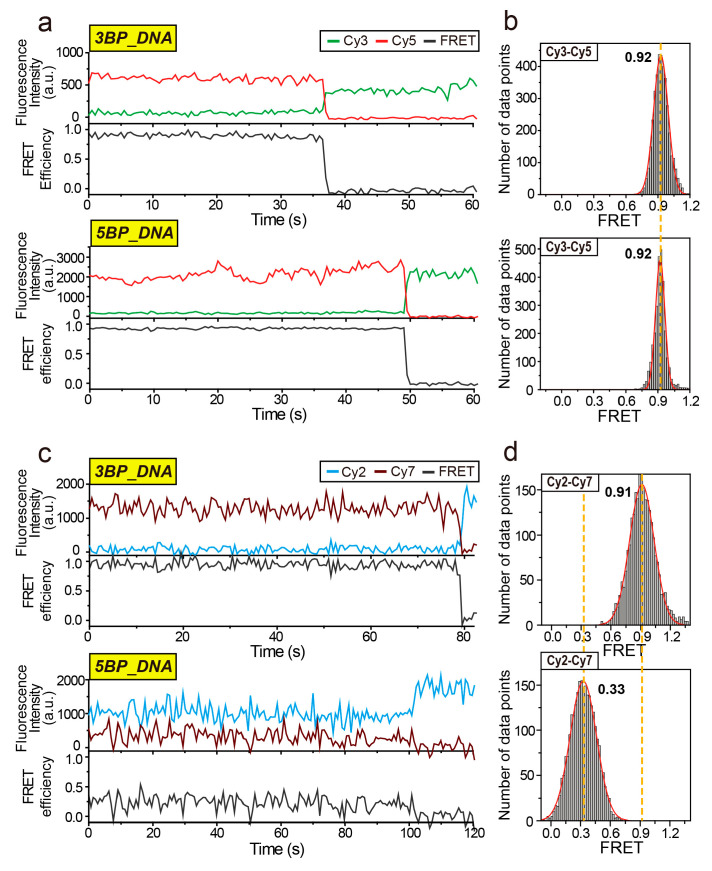
Single-molecule FRET measurement in proof-of-concept experiments using the double-stranded DNA constructs. (**a**) Representative time traces of Cy3 (green) and Cy5 (red) fluorescence, and the corresponding FRET efficiency (gray) for 3BP_DNA (upper graph) and 5BP_DNA (lower graph) constructs. (**b**) FRET histograms of the Cy3–Cy5 pair for 3BP_DNA (upper graph) and 5BP_DNA (lower graph). Average FRET efficiencies of the Cy3–Cy5 pair for the two DNA constructs were obtained by fitting the FRET histogram to a single Gaussian function. (**c**) Representative time traces of Cy2 (blue) and Cy7 (purple) fluorescence, and the corresponding FRET efficiency (gray) for 3BP_DNA (upper graph) and 5BP_DNA (lower graph) constructs. (**d**) FRET histograms of the Cy2–Cy7 pair for 3BP_DNA (upper graph) and 5BP_DNA (lower graph). Average FRET efficiencies of the Cy2–Cy7 pair for the two DNA constructs were obtained by fitting the FRET histogram to a single Gaussian function. FRET histograms were obtained by taking the FRET efficiency of 10 s for each molecule out of more than 100 molecules, except photobleaching events. The single-step intensity drop of the acceptor and rise of the donor in the representative intensity traces demonstrate that the measured fluorescence signals are derived from a single donor and acceptor pair.

Figure 3a,c show representative time traces of fluorescence intensity and FRET efficiency from the two DNA constructs (upper graph for 3BP_DNA, lower graph for 5BP_DNA) labeled with the Cy3–Cy5 pair (Figure 3a) or Cy2–Cy7 pair (Figure 3c). As shown in the traces, despite concerns about fluctuations of fluorescence intensity in close proximity between donor and acceptor fluorophores [20,21], both FRET pairs showed quite stable FRET signals, even at a short distance of 1.10 nm. First, in the conventional Cy3–Cy5 pair, similar high FRET states (~0.92) were observed in the two DNA constructs, indicating that the inter-dye distances of these two constructs are beyond the working range of the Cy3–Cy5 pair. However, in the Cy2–Cy7 pair, we clearly observed a dramatic difference in FRET efficiency between these two DNA constructs, verifying distinguishability below 3 nm of the Cy2–Cy7 pair as predicted. Owing to relatively lower dye photostability, in this measurement, we expected a considerable reduction in the signal-to-noise of the Cy2–Cy7 pair compared to that of the Cy3–Cy5 pair. Nonetheless, we were able to observe sub-nanometer distance changes at a short distance range.

For quantitative understanding of inter-dye distance changes, we obtained FRET histograms of the two DNA constructs for the Cy3–Cy5 pair (Figure 3b) or Cy2–Cy7 pair (Figure 3d), which consistently indicates that the two DNA constructs were distinguishable in the Cy2–Cy7 pair but not in the Cy3–Cy5 pair. Using the measured FRET efficiencies and *R_0_* value of Cy2–Cy7, we determined that the inter-dye distances for 3BP_DNA and 5BP_DNA are 1.49 and 2.48 nm, respectively, which confirms the ability of the Cy2–Cy7 pair to explore the short distance range with high accuracy. These results are in good agreement with the values predicted by the structural model prediction, supporting the conclusion that the single-molecule FRET method using the Cy2–Cy7 pair enables us to accurately observe the subtle conformational changes even at a short distance range.

## 4. Conclusions

In this work, we demonstrated a single-molecule FRET assay that can observe the small conformational changes of biomolecules at a short distance range below the lower limit of conventional FRET pairs by using the Cy2–Cy7 pair, of which the spectral overlap between donor emission and acceptor absorption is extremely small. In contrast to the other methods for monitoring short distance changes, our approach is readily available for single-molecule FRET applications by replacing a conventional FRET pair with the Cy2–Cy7 pair. A proof-of-concept experiment using a cylindrical model for DNA duplexes showed that our method can reliably observe distance changes at a short distance range. We anticipate that our single-molecule FRET assay proposed here will be useful for describing the subtle conformational dynamics of protein domains with advanced labeling techniques [22,23,24]. Thus, our method opens up new opportunities for understanding the dynamic nature of various proteins.
